# Clinical Significance of a Novel Vasculogenic Mimicry-based Prognostic Model in Hepatocellular Carcinoma

**DOI:** 10.2174/0109298673298862240510073543

**Published:** 2024-05-16

**Authors:** Yifan Zeng, Shuwen Jiang, Zhuoqi Lou, Lin Chen, Yongtao Zhang, Liya Pan, Qingmiao Shi, Bing Ruan

**Affiliations:** 1 State Key Laboratory for Diagnosis and Treatment of Infectious Diseases, National Clinical Research Center for Infectious Diseases, National Medical Center for Infectious Diseases, Collaborative Innovation Center for Diagnosis and Treatment of Infectious Diseases, The First Affiliated Hospital, Zhejiang University School of Medicine, Hangzhou City, 310003, China

**Keywords:** Hepatocellular carcinoma, vasculogenic mimicry, machine learning, prognostic prediction model, tumor microenvironment, immunotherapy

## Abstract

**Background:**

Vasculogenic mimicry, a novel neovascularization pattern of aggressive tumors, is associated with poor clinical outcomes.

**Objective:**

The aim of this research was to establish a new model, termed VC score, to predict the prognosis, Tumor Microenvironment (TME) components, and immunotherapeutic response in Hepatocellular Carcinoma (HCC).

**Methods:**

The expression data of the public databases were used to develop the prognostic model. Consensus clustering was performed to confirm the molecular subtypes with ideal clustering efficacy. The high- and low-risk groups were stratified utilizing the VC score. Various methodologies, including survival analysis, single-sample Gene Set Enrichment Analysis (ssGSEA), Tumor Immune Dysfunction and Exclusion scores (TIDE), Immunophenoscore (IPS), and nomogram, were utilized for verification of the model performance and to characterize the immune status of HCC tissues. GSEA was performed to mine functional pathway information.

**Results:**

The survival and immune characteristics varied between the three molecular subtypes. A five-gene signature (TPX2, CDC20, CFHR4, SPP1, and NQO1) was verified to function as an independent predictive factor for the prognosis of patients with HCC. The high-risk group exhibited lower Overall Survival (OS) rates and higher mortality rates in comparison to the low-risk group. Patients in the low-risk group were predicted to benefit from immune checkpoint inhibitor therapy and exhibit increased sensitivity to immunotherapy. Enrichment analysis revealed that signaling pathways linked to the cell cycle and DNA replication processes exhibited enrichment in the high-risk group.

**Conclusion:**

The VC score holds the potential to establish individualized treatment plans and clinical management strategies for patients with HCC.

## 1 INTRODUCTION

Primary liver cancer ranks as the sixth most prevalent malignancy globally [1]. In 2020, there were 905,700 diagnoses and 830,200 deaths attributed to primary liver cancer worldwide. Hepatocellular Carcinoma (HCC), a dominant histological subtype within primary liver cancer, constitutes approximately 75% of all diagnosed liver cancer cases. The 5-year survival rate of patients with HCC is < 20% as the malignancy is diagnosed at an advanced stage [2]. Surgical interventions, including liver resection and transplantation, are the primary treatment for HCC [3]. Furthermore, the Food and Drug Administration has granted approval for six systemic therapies for HCC [4]. Among these, lenvatinib and sorafenib serve as first-line drugs in systemic therapy for HCC. However, the beneficial effects of these conventional therapies are limited to the early stage of HCC. Past research has demonstrated the significance of the Tumor Microenvironment (TME) in the onset and progression of HCC. Furthermore, a correlation has been observed between the components of the TME and the prognosis of patients with HCC. However, reliable prognostic biomarkers for HCC have not been identified. As the progression of HCC is regulated by the immune system, immunotherapies, such as immune checkpoint inhibitors, virotherapy, and adoptive T-cell transfer, are considered innovative therapeutic modalities [5]. Hence, there is a pressing need to identify biomarkers that can predict the clinical outcomes and treatment responses in patients with HCC.

Vasculogenic Mimicry (VM), a phenomenon exhibited by invasive tumor cells, represents a phenotype of Epithelial-mesenchymal Transition (EMT), which occurs in epithelial-derived tumor cells triggered by hypoxia and angiogenic factors [6, 7]. VM is the formation of microvascular channels in the extracellular matrix that transport fluid independent of endothelial vessels [8]. During the development of VM, the expression levels of some epithelial cell markers, including CDH1, CTNNA1, and TJP1, are downregulated, whereas those of mesenchymal cell markers, such as CDH5 and CDH2, are upregulated. EMT plays a crucial function in VM-forming tumor cells and promotes the invasion and metastasis of tumors through diverse mechanisms [9]. Various signaling pathways are involved in VM. In particular, the miR200-ZEB1 and miR34-SNAIL1 axes are considered the major regulators of VM formation. The miR200-ZEB1 and miR34-SNAIL1 axes downregulate the transcription of CDH1 to suppress the epithelial properties of cells, contributing to the disruption of cell-to-cell adhesions and consequently promoting tumor metastasis. In addition, VM is associated with poor clinical outcomes. Prior research has identified VM in diverse cancer types, including HCC, melanoma, and ovarian cancer [10].

The invasiveness of HCC, a typical hyper-vascular solid tumor, is closely associated with microvascular invasion. The vascular network, including VM, holds significance in supporting the rapid growth of tumors by ensuring an ample supply of oxygen and nutrients [11]. Earlier research has shown a positive correlation between the expression of Methyltransferase-like 3 (METTL3) and VM formation in HCC tissues *via* the Hippo-YAP1 pathway [10]. Ou *et al.* revealed that VM induced by Frizzled 2 (FZD2) may be a key step in the metastasis and early recurrence of HCC [12]. Besides, hypoxia-related VM mediated by integrins A5 and B1 (ITGA5 and ITGB1) could be one of the reasons for sorafenib resistance in HCC patients [13]. Previous studies have identified the presence of VM in HCC and its association with poor clinical prognosis [9, 14, 15]. However, few studies have developed prognostic models based on VM-related genes and the principles of machine learning.

The aim of the research was to establish a new HCC prognostic model using public transcriptomics databases. Patients with HCC were stratified based on VM-related genes. The clinicopathological, immunological, and pathway characteristics of the stratified patients were subjected to comparative analysis. Then, a prognostic model (defined as VC score) was constructed based on VM-derived subtypes, and validated using testing and immunotherapy cohorts to prove that the model had high accuracy in predicting survival status and treatment response. The findings of this research provide useful insights for developing novel management and personalized therapeutic strategies for patients with HCC.

## 2 MATERIALS AND METHODS

### 2.1 Data Extraction and Preprocessing

Clinical information and RNA sequencing data of HCC from The Cancer Genome Atlas-Liver Hepatocellular Carcinoma (TCGA-LIHC) dataset were utilized for the establishment of a prognostic model. Validation cohorts included the International Cancer Genome Consortium-Japanese Liver Cancer (ICGC-LIRI-JP) and GSE14520 datasets. The latest expression statistics and clinical follow-up information, comprising RNA-Seq (FPKM) files of patients with HCC, were obtained from the TCGA database. TCGA-LIHC cohort included 371 tumor samples and 50 paracancerous control samples. After removing the data of samples without information on survival status and time, the information of 365 HCC samples was obtained. The ICGC-LIRI-JP dataset (n = 203) was obtained from the ICGC database. Additionally, the GSE14520 dataset (n = 221) was acquired from the Gene Expression Omnibus database. VM-related genes (n = 182) were retrieved from prior research [16].

### 2.2 Consensus Clustering

Consensus clustering analysis of VM-related Differentially Expressed Genes (DEGs) associated with prognosis was performed using the ConsensusClusterPlus package to construct a consistent matrix and classify the samples. The following parameters were used: clusterAlg=”km”; distance=”Euclidean”; 500 replications with a sampling proportion of 80% each time.

The samples were classified using consensus clustering. A Cumulative Distribution Function (CDF) plot was employed to determine the optimal cluster number. The CDF delta area curve yielded stable clustering results, and the ideal number of clustering classifications was determined to be three.

### 2.3 Construction of Risk Models

DEGs across the various subtypes in the TCGA cohort were identified utilizing the “limma” package based on the mentioned criteria: False Discovery Rate (FDR) < 0.05 and |log2 Fold Change (FC)| > 1; *p* < 0.05. These DEGs underwent univariate Cox regression analysis, after which the Least Absolute Shrinkage and Selection Operator (LASSO) regression analysis was conducted to decrease the gene number for constructing the risk model with R “glmnet” [17]. The VC score was evaluated as mentioned below:

VC score = Σβi × Expi.

Patients were stratified into high and low-risk groups as per the median VC score. The survival rate was evaluated utilizing the Kaplan-Meier (K-M) method. Additionally, time-dependent Receiver Operating Characteristic (ROC) curves were generated.

### 2.4 Landscape of Cell Infiltration in TME

The data on immunocytes associated with both innate immunity and adaptive immunity were sourced from prior research [18]. Immune function was evaluated *via* R “GSVA”. The gene markers of 27 immune signatures were downloaded from previous studies [19-21]. Single-sample Gene Set Enrichment Analysis (ssGSEA) was performed to investigate the abundance of immunocytes.

Estimation of Stromal and Immune cells in Malignant Tumors using Expression data (ESTIMATE) is an algorithm that infers the matrix and immune scores in tumor tissues based on ssGSEA [22]. This research made use of ESTIMATE to examine the infiltrating stromal and immune cell levels.

### 2.5 Nomogram Development based on Clinicopathological Features

A nomogram was developed utilizing R “rms”, incorporating the risk score and American Joint Committee on Cancer (AJCC) stage. This nomogram serves as a quantitative and reliable analytical tool for predicting the Overall Survival (OS) of patients with HCC. To evaluate its accuracy, a calibration curve was generated.

### 2.6 Evaluation of Immune Therapy Response

Tumor Immune Dysfunction and Exclusion (TIDE), which is a computational framework that provides information on tumor cell dysfunction, was used for predicting the clinical treatment response to immunotherapy [23].

Immunophenoscore (IPS) was calculated to evaluate the differential immune treatment responses using the IOBR package [24]. Four immunophenotypes (antigen presentation, effector cells, suppressor cells, and checkpoint markers) were quantified with diverse immune biomarkers. IPS represents the overall score and can predict immunotherapeutic efficacy. The IPS value is positively correlated with the immunogenicity of samples.

The IMvigor210 cohort is an immune therapy cohort of late metastatic urothelial carcinoma. The gene expression profile and clinical information from the IMvigor210 cohort were incorporated to identify independent prognostic factors for HCC and estimate the predictive capability of the risk score for immunotherapeutic sensitivity.

### 2.7 Functional Enrichment Analysis

GSEA was conducted utilizing the “clusterProfiler”, and the analysis was based on the “h.all.v7.5.1.symbols.gmt” gene set [25]. Pathways were deemed significant if they exhibited a *p* value < 0.05 and FDR < 0.05.

### 2.8 Cell Culture

The hepatoma cell line (HepG2 cells) and the healthy hepatocyte cell line (LO2 cells) were sourced from the Chinese Academy of Sciences (Shanghai, China). HepG2 cells were grown in Dulbecco’s modified Eagle’s medium (Gibco, USA), while Roswell Park Memorial Institute-1640 medium was utilized for LO2 cells (Gibco, USA). Both media were supplemented with 10% fetal bovine serum (Gibco, USA) and 1% penicillin/streptomycin (Beyotime, China). The cultures were kept at 37°C under 5% CO_2_ in an incubator.

### 2.9 RNA Extraction and Quantitative Real-time Polymerase Chain Reaction (qRT-PCR)

Total RNA was extracted from the cells employing the RNA easy mini kit (QIAGEN, China), following the provided protocol. The RNA then underwent reverse transcription into complementary DNA utilizing the PrimeScript RT master kit (Takara, Japan). TB Green Premix (Takara, Japan) was used for DNA amplification. The relative mRNA expression levels were determined utilizing the 2^−ΔΔCT^ method. GAPDH was utilized as the internal reference. The primers are given in Table **S1**.

### 2.10 Statistical Analysis

The research employed a range of statistical analyses, including Spearman correlation analysis, univariate/multivariate Cox regression analysis, and ANOVA test. Student's t-test was used to assess the difference in relative mRNA expression levels between the hepatoma cell line and the hepatocyte line. These analyses were carried out employing R 3.6.0 and GraphPad Prism 8.0.1, with differences deemed significant at a threshold of *p* < 0.05.

## 3 RESULTS

### 3.1 Consensus Clustering of VM-related Genes

**Fig (1):**
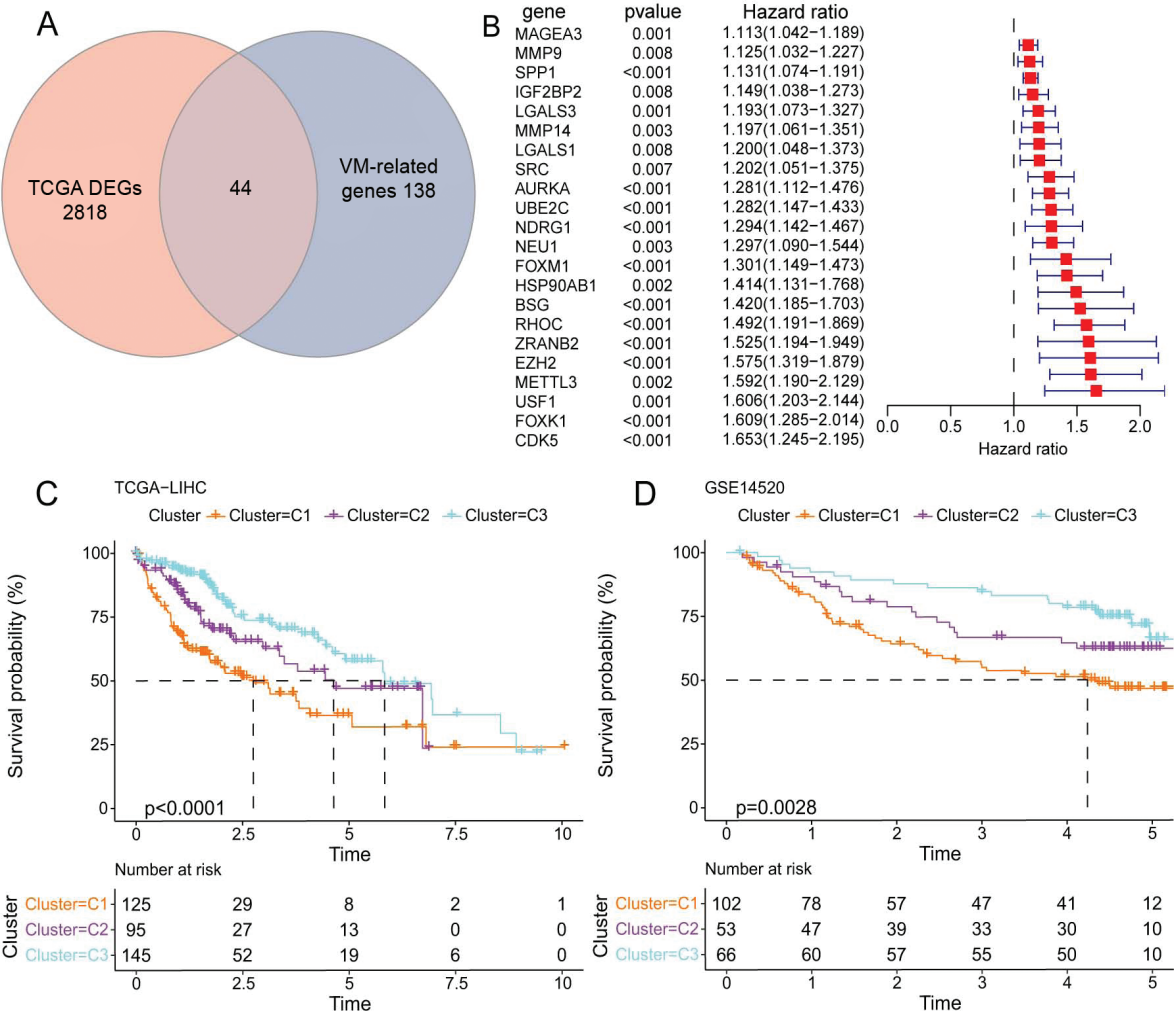
Consensus clustering of patients with Hepatocellular Carcinoma (HCC) based on Vasculogenic Mimicry (VM)-related genes. (**A**) The intersection of Differentially Expressed Genes (DEGs) identified from the Cancer Genome Atlas-Liver Hepatocellular Carcinoma (TCGA-LIHC) cohort with VM-related genes. (**B**) 22 prognosis-related genes. (**C**) K-M survival analysis of TCGA-LIHC cohort. (**D**) K-M survival analysis of the GSE14520 cohort.

Analysis of the data of HCC and adjacent non-cancerous samples in the TCGA-LIHC cohort revealed 2862 DEGs. Next, 182 VM-related genes were intersected with 2862 DEGs to obtain 44 genes (Fig. **1A**). Then, 22 prognosis-related genes (*p* < 0.01), such as SPP1, EZH2, METTL3, USF1, FOXK1, and CDK5, were identified from univariate Cox regression analysis (Fig. **1B**). Consensus clustering analysis of 22 VM-related DEGs in TCGA-LIHC cohort revealed three molecular subtypes of HCC (Fig. **1C**). The prognosis of Cluster 3 (C3) could be distinguished from that of Cluster 1 (C1), which was also verified in the external validation dataset GSE14520 (Fig. **1D**).

### 3.2 VM-related Subtypes had Distinct Immunogenomic Patterns and Dysregulated Pathways

**Fig (2):**
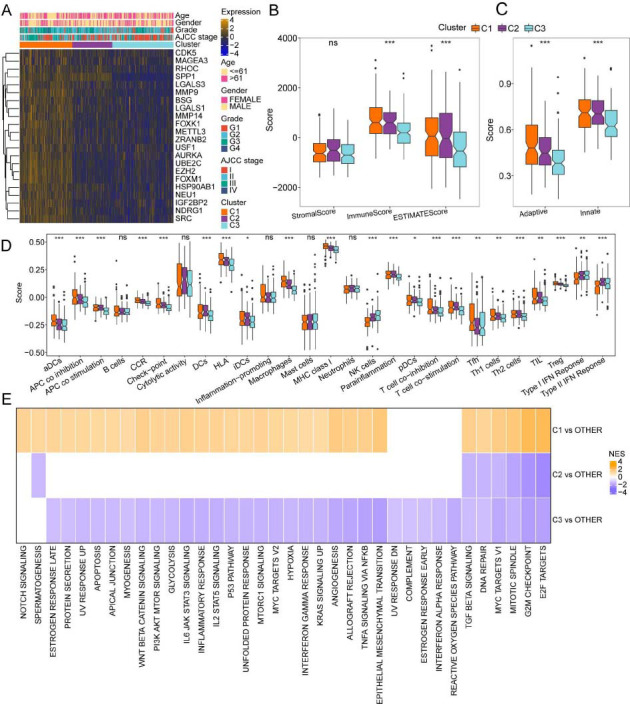
Immune and signaling pathway characteristics of the three molecular subtypes. (**A**) Differential gene expression levels and clinical characteristics between molecular subtypes. (**B**) Estimation of Stromal and Immune cells in Malignant Tumors using Expression data (ESTIMATE) scores. (**C**) Innate immunity and adaptive immunity scores. (**D**) Comparison of 27 immune components evaluated using single-sample Gene Set Enrichment Analysis (ssGSEA). (**E**) Gene Set Enrichment Analysis (GSEA) of differentially expressed genes between the molecular subtypes.

The clinicopathological characteristics, encompassing age, gender, grade, and AJCC stage, were comparatively analyzed among patients with HCC in the C1, C2, and C3 subtypes. The C1 subtype exhibited a higher proportion of patients with advanced-stage cancer, which was associated with poor prognosis. Moreover, the C1 subtype showed upregulated expression levels of DEGs related to VM (Fig. **2A**). To elucidate the differential immune characteristics across different subtypes, the TME was examined using various published methodologies. As shown in Figs. (**2B**-**C**), the immune/ESTIMATE scores and adaptive/innate scores were high in the C1 subtype. Subsequent analysis of immune infiltration unveiled an upregulation in the infiltration levels of various immunocytes, such as Dendritic Cells (DCs), follicular helper T cells (Tfh), macrophages, T helper 1 (Th1) cells, Th2 cells, and regulatory T cells (Tregs) in the C1 subtype. Furthermore, immune functions, including Antigen-presenting Cell (APC) co-inhibition, checkpoint activity, Major Histocompatibility Complex (MHC) class I expression, and T-cell co-inhibition, were found to be upregulated in the C1 subtype. The infiltration levels of Natural Killer (NK) cells and the type I/II Interferon (IFN) response were upregulated in the C3 subtype (Fig. **2D**). Moreover, functional enrichment analysis was conducted utilizing GSEA (p.adjust < 0.05). In comparison to the C2 and C3 subtypes, the expression levels of genes related to pathways, such as E2F targets, G2M checkpoints, and mitotic spindle MYC targets, were found to be upregulated in the C1 subtype (Fig. **2E**). These findings indicated that tumor cells in the C1 subtype exhibited a strong proliferative potential and that the mechanism underlying immune escape of C1 subtype may be related to the downregulation of immunogenicity.

### 3.3 Establishment of the VC Score Model for the Prediction of the Prognosis of Patients with HCC

**Fig (3):**
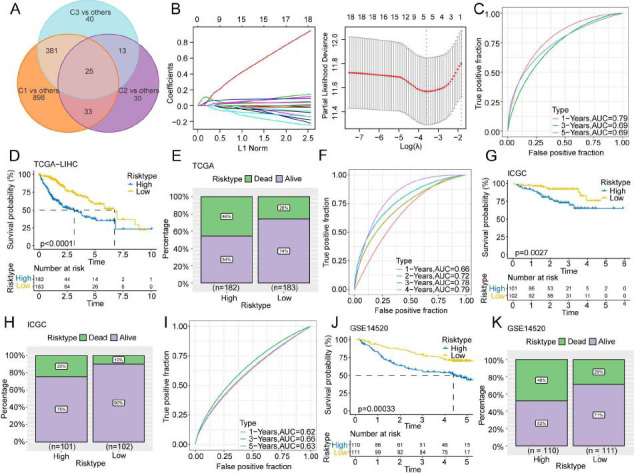
Establishment and validation of the risk model (VC score). (**A**) Venn diagram of Differentially Expressed Genes (DEGs). (**B**) Least absolute shrinkage and selection operator and Cox regression analyses were executed to reduce the number of genes. (**C**-**K**) Receiver Operating Characteristic (ROC) curve, K-M survival curves, and survival status of TCGA-LIHC (**C**-**E**), International Cancer Genome Consortium-Japanese Liver Cancer (ICGC-LIRI-JP) (F-H), and GSE14520 (**I**-**K**) cohorts.

The DEGs between the following pairs were identified employing the limma package: C1 subtype and other subtypes; C2 subtype and other subtypes; C3 subtype and other subtypes (FDR < 0.05; |log2FC| >1). These DEGs were intersected to obtain 25 overlapping genes (Fig. **3A**). Univariate Cox analysis identified 19 genes that could affect the prognosis of patients with HCC (*p* < 0.05). Furthermore, the LASSO regression analysis of these 19 genes was carried out to decrease the number of prognostic genes. This resulted in the identification of the following five key genes: TPX2, CDC20, CFHR4, SPP1, and NQO1. These five genes were employed for the construction of the risk scoring system (VC score) (Fig. **3B**). The VC score was calculated using the following formula:

VC score = [(0.21 × TPX2 expression level) + (0.048 × CDC20 expression level) + (−0.053 × CFHR4 expression level) + (0.075 × SPP1 expression level) + (0.044 × NQO1 expression level)]

The VC scores of all the patients were calculated based on the expression level of the genes and the risk coefficient. The prognostic value of the VM-derived risk score was examined in the TCGA-LIHC cohort. Taking the median value of the VC score into account (1.50527), patients with HCC were stratified into high-risk (n = 182) and low-risk (n = 183) subgroups. Time-dependent ROC analysis implied that the model had a high area under the ROC curve value, suggesting a high prognostic accuracy of the VC score model (Fig. **3C**). K-M survival analysis demonstrated that the high-risk group exhibited a lower OS in comparison to the low-risk group (*p* < 0.0001) (Fig. **3D**). The mortality rates (46%) in the high-risk group were considerably elevated relative to the low-risk group (26%) (Fig. **3E**). The prediction performance of the VC score model in the ICGC-LIRI-JP (Figs. **3F**-**H**) and GSE14520 (Figs. **3I**-**K**) cohorts was similar to that in the training cohort. The AUC values of 0.72, 0.78, and 0.79 for 2, 3, and 4 years in the ICGC-LIRI-JP cohort confirmed the feasibility of the VC score.

### 3.4 VC Score Served as an Independent Prognostic Factor to Construct the Nomogram

**Fig (4):**
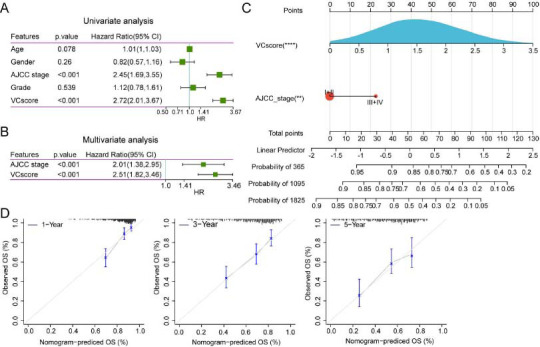
Construction and identification of nomogram. (**A**-**B**). Results of univariate and multivariate Cox regression analyses. (**C**) Nomogram constructed with American Joint Committee on Cancer (AJCC) stage and VC score. (**D**) The calibration curve of the nomogram model.

To optimize the model, VC score and clinical features (age, gender, AJCC stage, and tumor grade) were included in the univariate and multivariate Cox regression analyses. VC score [*p* < 0.001, Hazard Ratio (HR) = 2.51, 95% Confidence Interval (CI): 1.82-3.^46^] and AJCC stage (*p* < 0.001, HR = 2.01, 95% CI:1.38-2.95) were determined to be independent predictive factors for the prognosis of patients with HCC (Figs. **4A**-**B**). To quantify and visualize the risk assessment and survival probability of patients, the VC score and AJCC stages were combined to establish a nomogram. VC score exhibited enhanced performance in predicting the survival rate (Fig. **4C**). The calibration curve demonstrated the prediction accuracy of the VC score model (Fig. **4D**). The predicted calibration curves of the 1-year, 3-year, and 5-year OS of patients were close to the standard curve, suggesting the favorable prediction performance of the nomogram.

### 3.5 Differential TME Landscapes among the Risk Groups

**Fig (5):**
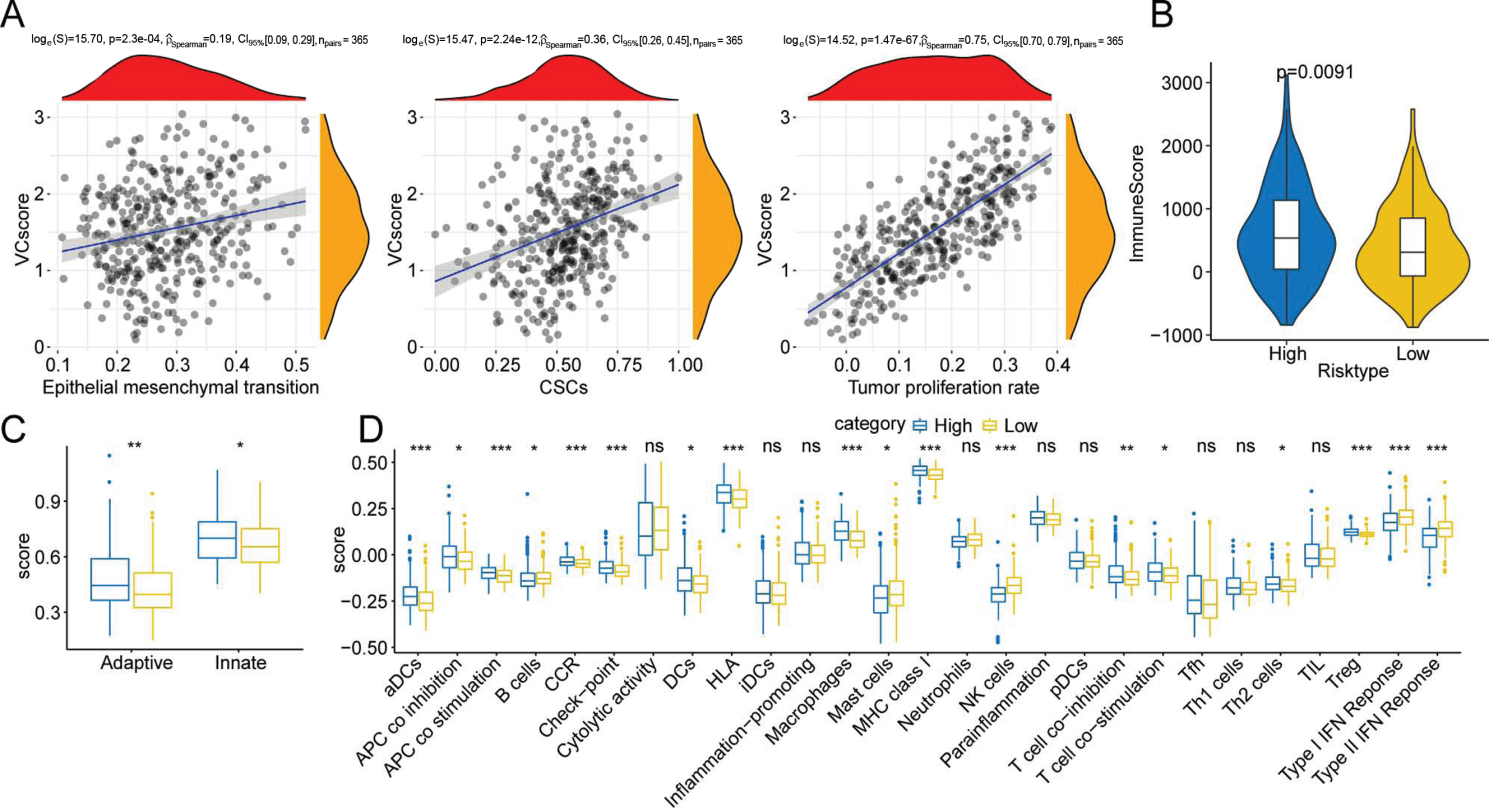
Prediction of differential immune landscapes across high and low-risk groups. (**A**) Scatter plot of the correlation between VC score and Epithelial-mesenchymal Transition (EMT), Cancer Stem Cells (CSCs), and tumor proliferation rate. (**B**) The immune score was derived through the ESTIMATE algorithm. (**C**) Innate immunity and adaptive immunity scores. (**D**) Comparison of 27 immune components.

To determine the potential pathways associated with VC score, the correlation between VC score and VM formation-related mechanisms, including EMT, Cancer Stem Cells (CSCs), and tumor proliferation rate, was examined (Fig. **5A**). VC score was positively related to the tumor proliferation rate (Spearman's rho = 0.75, *p* < 0.001). To further clarify the differential TME landscapes across both risk groups, the infiltration levels of immunocytes in HCC tissues were examined in the TCGA-LIHC cohort. The immune and adaptive/innate scores were elevated in the high-risk group, implying a high overall immune level in the TME (Figs. **5B**-**C**). ssGSEA of 27 immune signatures revealed that the abundance of activated DCs, macrophages, DCs, Th2 cells, and Tregs was upregulated in the high-risk group. Meanwhile, the abundance of various immunocytes, encompassing B cells, NK cells, and mast cells, was upregulated in the low-risk group. The activity of some immune functions and some immune components, including APC co-inhibition, chemokine receptors, APC co-stimulation, checkpoint, human lymphocyte histocompatibility antigen, T-cell co-inhibition, MHC class I, and T-cell co-stimulation, was upregulated, whereas that of type I/II IFN response was downregulated in the high-risk group (Fig. **5D**).

### 3.6 Immunotherapeutic Benefits Predicted by the VC Score

**Fig (6):**
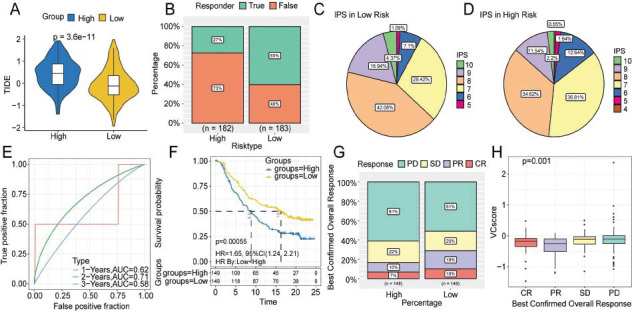
Prediction of immunotherapy response using the VC score. (**A**-**B**). Differential Tumor Immune Dysfunction and Exclusion (TIDE) scores and response ratios. (**C**-**D**) The fan diagram shows the differential Immunophenoscores (IPSs) between high- and low-risk groups. (**E**-**F**) ROC and K-M curves of the IMvigor210 cohort. (**G**-**H**) The relationship between VC score and efficacy of immunotherapy. **Abbreviations: ** CR, complete response; PR, partial response; SD, stable disease; PD, progressive disease.

Afterward, the ability of VC score to predict the response to immunotherapy was examined. The TIDE scores among the subgroups were comparatively analyzed to assess potential immune dysfunction in HCC. Compared to those in the high-risk group, the TIDE score was decreased (Fig. **6A**) and the response to immunotherapy (60% *vs.* 27%) was higher in the low-risk group (Fig. **6B**). This indicated that patients with HCC in the low-risk group can benefit from immune checkpoint inhibitor therapy. IPS was used as a common indicator to evaluate the immunogenicity of samples. The proportion of HCC samples with a high IPS was upregulated in the low-risk group, indicating the patients in this group to be sensitive to immunotherapy (Figs. **6C**-**D**). In the IMvigor210 immunotherapy cohort, survival analysis revealed that patients with a high VC score were associated with short survival time (HR = 1.65; 95% CI = 1.24-2.21; Figs. **6E**-**F**). Inhibiting immune checkpoints utilizing monoclonal antibodies that function by blocking the T-cell suppressor PD-L1 is a novel and potent anti-cancer treatment [26]. Hence, data from patients undergoing anti-PD-L1 immunotherapy in the IMvigor210 cohort were used to predict treatment response. Compared to that in the high-risk group, the proportion of patients with Complete Response (CR) and Partial Response (PR) was higher in the low-risk group (Fig. **6G**). The values of the VC score in the stable disease/progressive disease group were higher in comparison to the CR/PR group (Fig. **6H**). This outcome implied that the novel prognostic biomarker VC score can predict the results of immunotherapy.

### 3.7 Biological Pathways and Functional Enrichment Analysis

**Fig 7:**
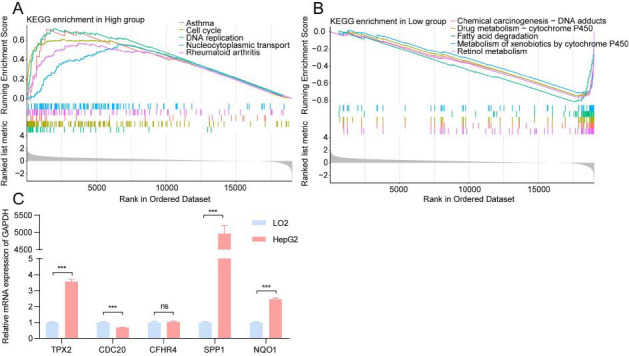
Enrichment of biological functions and the results of quantitative Real-time Polymerase Chain Reaction (qRT-PCR). (**A**-**B**) The results of GSEA in different risk subgroups. (**C**) The expression levels of five signature genes that were used to construct the VC score.

GSEA was conducted for further clarification of the signaling pathways enriched in the risk subgroups. Five pathways with the highest normalized enrichment score were chosen for visualization. Cell cycle and DNA replication signaling pathways were enriched in the high-risk group (Fig. **7A**), while some metabolism-related pathways, including fatty acid degradation, retinol metabolism, drug metabolism, and xenobiotics metabolism by cytochrome P450 were enriched in the low-risk group (Fig. **7B**). Thus, cell cycle regulation and metabolic activity may be the potential mechanisms through which VC score can predict the prognosis of patients with HCC.

## 3.8 *In Vitro* Experimental Validation of Signature Genes

The expression levels of five signature genes of the VC score model in hepatoma cells (HepG2 cells) and healthy hepatocytes (LO2 cells) were analyzed employing qRT-PCR. The TPX2, SPP1, and NQO1 mRNA levels in HepG2 cells were higher in comparison to those in LO2 cells (Fig. **7C**), and were in line with the outcomes of bioinformatics analysis. TPX2, SPP1, and NQO1, which are reported to be oncogenes, are involved in tumorigenesis, immunity, and metabolism [27-30]. However, the CFHR4 expression levels lacked any remarkable variation when assessed between HepG2 and LO2 cells, which can be attributed to the limitations of cell-level experiments. In summary, the results of *in vitro* experiments supported our novel prognostic model.

## 4 DISCUSSION

HCC, a common carcinoma of the digestive system, is one of the major contributors to cancer-associated fatalities globally [31]. The progression of HCC is rapid with patients being generally diagnosed with middle-stage and advanced-stage tumors. Hence, the efficacy of conventional treatment for HCC is poor. Metastasis and recurrence are the key factors affecting the survival of patients with HCC. Recently, breakthroughs have been achieved in the treatment of HCC, including locoregional therapies and molecular therapies [32]. However, clinical decisions on HCC treatment and the selection of personalized treatment are challenging as effective methods are not available to stratify patients with HCC according to their prognosis.

The invasiveness of HCC, a typical hyper-vascular solid tumor, is closely related to microvascular invasion [15]. Research from the past two decades has suggested that the presence of VM, which enables progressive tumors to enter the blood circulation, in HCC tissues, may be correlated with the invasion and metastasis potential and adverse clinical prognosis of the disease [33, 34]. Alongside EMT and CSCs, the formation of VM is regulated by various signaling molecules, such as HIF1A, matrix metalloproteinase, PTK2, PIK3CA, and CDH5 [35-38]. The mechanism underlying VM formation involves the transformation of tumor cell genotype, the interaction between tumor cells and extracellular matrix, and the modification of molecular signal. Hence, a reliable prognostic model can be developed based on VM-related genes. This research combined such genes with clinical information to establish a prognostic model with predictive value for patients with HCC, providing valuable insights for clinical management.

This research first identified 22 VM-related molecular markers, such as SPP1, EZH2, METTL3, USF1, FOXK1, and CDK5 using high-throughput sequencing data from the TCGA-LIHC dataset and VM-related genes. Past research has reported that the m6A methyltransferase METTL3 is upregulated in HCC tissues and promotes VM formation through the Hippo-YAP1 axis, enhancing the migratory and invasive capacities of HCC cells *in vitro* and *in vivo* [39]. Thus, VM formation is linked to adverse prognostic outcomes in patients [10]. Yao *et al*. performed spatial transcriptomics and revealed that the hypoxic microenvironment promotes the expression of SPP1. SPP1^+^ Tumor-associated Macrophages (TAMs) and cancer-related fibroblasts, which are immunosuppressive cells in the Tumor Immune Microenvironment (TIME) of HCC, form a Tumor Immune Barrier (TIB) [29]. The TIB limits the infiltration of immune cells in the tumor center and suppresses the effect of immune checkpoint blockade, contributing to poor prognosis. A 2023 study reported the presence of SPP1-expressing TAM in alpha-fetoprotein-positive HCC tissues and demonstrated that SPP1 can inhibit the anti-cancer activity of T cells by binding to CD44 [40]. Other VM-related DEGs identified in this study have also been reported to be involved in the growth, migration, and stemness regulation of HCC and are closely correlated with the survival outcome of patients [41-43].

Clustering analysis of 22 VM-related DEGs revealed three molecular subtypes (C1, C2, and C3) of HCC. The survival time, clinicopathological features, and immune infiltration status varied across the three subtypes. The prognosis of the C1 subtype was worse. The C1 subtype was enriched in DNA replication pathways, such as E2F targets, G2M checkpoints, and mitotic spindle MYC targets. Immune infiltration analysis revealed that the tumor cells in the C1 subtype exhibited strong proliferation ability and immune evasion owing to the downregulation of immunogenicity.

Afterward, DEGs between the three molecular subtypes were identified. LASSO Cox regression analysis was executed after univariate Cox regression analysis to prevent overfitting. Five key genes (TPX2, CDC20, CFHR4, SPP1, and NQO1) were employed for the establishment of the novel prognostic VC score model. The five genes used to construct the VC score model have been reported to be closely correlated with tumorigenesis, and some of them have been used to construct prognostic models in previous studies [44, 45]. Fuqiang *et al*. reported that TPX2 could be stabilized by CDK5-mediated phosphorylation, promoting the proliferation, migration, and tumorigenicity of HCC cells [43]. NQO1 is upregulated in HCC cells and promotes EMT, cell proliferation, and angiogenesis through the NQO1/p53/SREBP1 axis [46]. Min *et al.* suggested that CDC20 regulates HIF1A activity by promoting the polyubiquitination and degradation of P3H3 and is associated with the OS of patients with HCC [47]. CFHR4, a secreted plasma protein synthesized by hepatocytes, is an important component of innate immunity with a critical role in the TIME [44]. The important role of SPP1 in the TME suggests the potential of VC score to predict immune infiltration. Thus, these key genes are strongly associated with VM and the prognosis of patients with HCC, validating their selection to build the prognostic model in this research.

The predictive performance of the VC score model was verified using the ICGC-LIRI-JP and GSE14520 datasets. These findings may aid in guiding the prognostic evaluation and predicting the pathological features of HCC. As per the median VC score value, patients with HCC were stratified into high- and low-risk groups. Survival analysis demonstrated the ability of VC score to predict the OS of patients as satisfactory. Multivariate Cox regression analysis and nomogram demonstrated VC score as an independent risk factor for the prognosis of patients with HCC. Correlation analysis demonstrated VC score as associated with EMT, CSCs, and tumor proliferation rate. The high- and low-risk groups exhibited different immune infiltration characteristics and immunotherapy responses. Furthermore, the ability of VC score to predict the sensitivity to immune checkpoint inhibitors was validated using the IMvigor210 immunotherapy cohort. Patients with HCC in the low-risk group were anticipated to experience positive outcomes from anti-PD-L1 immunotherapy. Functional enrichment analysis revealed that cell cycle regulation and metabolic activity may be the potential mechanisms through which VC score could predict the prognosis of patients with HCC. Additionally, the results of qRT-PCR analysis (at the cellular level) were consistent with those of bioinformatics analysis.

VM-related DEGs between molecular subtypes were identified in this investigation to establish a novel model for stratifying patients with HCC. The VC score, an independent prognostic indicator, exhibited excellent performance in predicting the prognostic outcomes, immunosuppressive status, and treatment response. This study, which analyzed public datasets, has involved some limitations. The performance of the VC score should be validated using a real-world clinical cohort. Moreover, additional research is warranted to elucidate the potential molecular mechanisms that underlie the correlation between VC score and HCC prognosis, either *in vivo* or *in vitro*.

## CONCLUSION

In conclusion, our study has identified three molecular subtypes (C1, C2, and C3) with different survival, clinicopathological, and signaling pathways and TIME features based on VM-related genes. A novel gene signature (TPX2, CDC20, CFHR4, SPP1, and NQO1) from the VM-derived subtypes has been constructed and used to provide valuable clinical references for HCC patients in predicting prognosis and immunotherapy effect.

## Data Availability

All data relevant to this study will be available from the corresponding author on special request. Public data can be found on the TCGA database (TCGA-LIHC) (https://www.cancer.gov/tcga), the ICGC database (ICGC-LIRI-JP) (https://dcc.icgc.org/), the GEO database (GSE14520) (https://www.ncbi.nlm.nih.gov/geo/), and the IMvigor210 cohort (https://research-pub.gene.com/IMvigor210CoreBiologies/).

## LIST OF ABBREVIATIONS

TME: Tumor Microenvironment
HCC: Hepatocellular Carcinoma
ssGSEA: Single-sample Gene Set Enrichment Analysis
TIDE: Tumor Immune Dysfunction and Exclusion
OS: Overall Survival
VM: Vasculogenic Mimicry
