# Supplementary material for: Clinical Significance of a Novel Vasculogenic Mimicry-based Prognostic Model in Hepatocellular Carcinoma
Source: Curr Med Chem. 2024 May 16;32(19):3926–40. doi: 10.2174/0109298673298862240510073543 (PMC12376130; doi:10.2174/0109298673298862240510073543)
Supplement: Supplementary file 1 [file CMC-32-19-3926_SD1.pdf]

Supplementary Material

Clinical Significance of a Novel Vasculogenic Mimicry-based Prognostic Model in Hepatocellular Carcinoma

Yifan Zeng[1,#], Shuwen Jiang[1,#], Zhuoqi Lou[1,#], Lin Chen[1], Yongtao Zhang[1], Liya Pan[1], Qingmiao Shi[1] and Bing Ruan[1,*]

[1]*State Key Laboratory for Diagnosis and Treatment of Infectious Diseases, National Clinical Research Center for Infectious Diseases, National Medical Center for Infectious Diseases, Collaborative Innovation Center for Diagnosis and Treatment of Infectious Diseases, The First Affiliated Hospital, Zhejiang University School of Medicine, Hangzhou City, 310003, China*

Supplementary Table1. The information of primers sequences for qRT-PCR.

Primer Name	Sequence (5'-3')	Base
TPX2-F	CTCCACTCGATTCCACTGCTAAA	23
TPX2-R	TGCCCAATGACAAAGCAAGAC	21
CDC20-F	TGCAGACATTCACCCAGCATC	21
CDC20-R	GCATCCACGGCACTCAGACA	20
CFHR4-F	GGGGATACCATTGAATTTATGTGTA	25
CFHR4-R	TCCACTATGCCTTCCCTACACA	22
SPP1-F	GTGCATACAAGGCCATCCC	19
SPP1-R	CCTGACTATCAATCACATCGGAAT	24
NQO1-F	GGTACAGTTTGGCTAGGTATCATTCA	26
NQO1-R	AAGTTAAGTCCCTTAGGGCAGGT	23
GAPDH-F	CGCTGAGTACGTCGTGGAGT	20
GAPDH-R	TGCTGATGATCTTGAGGCTGTTG	23
